# Towards a Dual Lateral Flow Nanobiosensor for Simultaneous Detection of Virus Genotype-Specific PCR Products

**DOI:** 10.1155/2018/7691014

**Published:** 2018-02-20

**Authors:** Dimitra K. Toubanaki, Evdokia Karagouni

**Affiliations:** Laboratory of Cellular Immunology, Department of Microbiology, Hellenic Pasteur Institute, 127 Vas. Sofias Ave., 11521 Athens, Greece

## Abstract

Nervous necrosis virus (nodavirus) has been responsible for mass mortalities in aquaculture industry worldwide, with great economic and environmental impact. A rapid low-cost test to identify nodavirus genotype could have important benefits for vaccine and diagnostic applications in small- and medium-scale laboratories in both academia and fish farming industry. A dual lateral flow biosensor for simultaneous detection of the most prevalent nodavirus genotypes (RGNNV and SJNNV) was developed and optimized. The dual biosensor consisted of two antibody-based test zones, indicative of each genotype, and a control zone. The positive signals were visualized by gold nanoparticles functionalized with anti-biotin antibody, and the detection was completed within 20 min. Optimization studies included antibody type and amount determination for test zone construction, gold nanoparticle conjugate type selection for high signal generation, and detection assay parameter determination. Following optimization, the biosensor was evaluated with healthy and RGNNV-nodavirus-infected fish samples. The proposed assay's cost was estimated to be less than 3 €, including the required reagents and biosensor. This work presents important steps towards making a dual lateral flow biosensor for nodavirus genotyping; further evaluation with clinical samples is needed before the test is appropriate for diagnostic kit development.

## 1. Introduction

Diseases of viral etiology have been wreaking havoc in the aquaculture industry, which is considered of strategic importance for Greek, European, and worldwide economies. Viral diseases often wipe out entire stocks within days of onset of infection with major economic and environmental costs [1, 2]. One such disease is viral nervous necrosis (VNN), also known as vacuolating encephalopathy and retinopathy (VER) or encephalomyelitis. VNN causes high mortalities in larvae and juveniles of 120 farmed and wild marine fish species, in geographically diverse areas including Europe, Australia, North America, and many parts of Asia. In many cases, the mortality rates may reach 100% within one week after infection, and even after recovering from the disease, the surviving fish are inclined to perform poorly [3–5].

Nervous necrosis virus (NNV), also known as nodavirus, has been recognized as the causative agent of VNN. Fish nodavirus belongs to the Nodaviridae family and the *Betanodavirus* genus. The virus is round shaped and nonenveloped, 23–25 nm in diameter with icosahedral structure. The virus genome is bisegmented; that is, it is formed by two positive-sense RNA molecules which are single-stranded (RNA1 and RNA2), while it does not contain a poly(A) sequence at the 3′ end [6]. The RNA1 sequence encodes an RNA-dependent RNA polymerase (RdRp) [7] while RNA2 encodes the capsid or coat protein [6]. A subgenomic transcript of RNA1, called RNA3, encodes two other nonstructural proteins (B1 and B2) [5]. Phylogenetic analysis of *Betanodaviruses* indicated the presence of four distinct clusters of isolates: SJNNV (Striped Jack), TPNNV (Tiger Puffer), BFNNV (Barfin Flounder), and RGNNV (Red-Spotted Grouper) [8].

Nodaviruses belonging to different genotypes have different host ranges [9], and a particular viral strain can infect specific fish species at different geographical locations [10]. Diverse optimal in vitro growth temperatures have been associated with different nodavirus genotypes [11], a fact that seems to correspond with different in vivo pathogenicities. Thus, host specificity can be directly related to the viral phenotype and/or genotype [12–14]. As suggested, specific nodavirus genotypes have particular host ranges with distinct geographic distributions, revealing the virus' ability to adapt to different water temperatures [14, 15]. As recorded in epidemiological studies, the RGNNV genotype can be found in various warm-water fish species, especially groupers and sea bass, having the widest geographic distribution. The BFNNV genotype can be detected in cold-water marine fish species, while the TPNNV genotype has been found in a few fish species [5]. Even though it was believed that the SJNNV genotype could infect only Japanese fish species, it was recently detected in South Europe aquaculture sites [16]. More specifically, nodavirus strains isolated from the Atlantic coast of South Europe or the Mediterranean basin were found to belong to both SJNNV and RGNNV genotypes. Moreover, the simultaneous occurrence of those genotypes in a single animal has been found by phylogenetic analysis, indicating either reassortment or dual viral infection of the fish [13, 17–19].

Analysis of nodavirus genetic variation would vastly benefit the rational development of effective vaccines and diagnostic reagents. Molecular methods such as polymerase chain reaction (PCR) are extensively used for nodavirus detection [20–23], yet they cannot distinguish the different genotypes, which is vital for a complete strategy to eliminate the nodavirus from aquacultures effectively. The golden standard for *Betanodavirus* genotype evaluation is sequencing [24, 25]. However, its routine use for genotype screening is difficult since the method requires specialized and expensive instrumentation and software, while it is time-consuming as well. Genotyping techniques as restriction fragment length polymorphism (RFLP) [26] and a combination of RT-PCR and blot hybridization [17] have also been proposed for discrimination of nodavirus RGGNV and SJNNV genotypes. Our research group has recently developed a tetra-primer allele-specific PCR-based methodology, for detection of the RGNNV and SJNNV genotypes, in a rapid, specific, and sensitive format [27, 28]. Tetra-primer PCR is an allele-specific PCR methodology which relies on the amplification of the genotype-specific products simultaneously in a single PCR run, using four primers: a pair of outer (external) primers and two internal primers that are genotype-specific [29]. The tetra-primer PCR method is promising for nodavirus genotyping by medium-size research laboratories and fish farms; however, the amplicons detection by agarose gel electrophoresis or real-time PCR instrumentation are limiting its application in the field with a low-cost format.

Lateral flow paper biosensors (LFB) provide a tool, which is ideal for sensitive, reproducible, and accurate detection of PCR products, in a rapid way, implanted successfully in research laboratory setups. LFBs are prefabricated paper strips containing dry reagents that are activated by applying a sample-containing solution. They are designed for disposable single use and for applications where an on/off signal is sufficient [30, 31]. Lateral flow biosensors have been used as the detection method for analytes including DNA, mRNA, miRNA, proteins, biological agents, and chemical contaminants [32]. Our research group has developed a lateral flow biosensor for nodavirus amplification product detection enabling rapid and accurate positive virus sample visualization [33].

To further facilitate nodavirus genotyping with a promising technique, the aim of the present study was the development and optimization of a dry-reagent lateral flow biosensor for simultaneous visual detection of two different nodavirus genotypes, namely RGNNV and SJNNV. The dry-reagent biosensor was prepared by selecting the proper antibodies and optimizing their deposited amounts. Next, gold nanoparticles, which serve as signal reporters, were modified by conjugation with anti-biotin antibody. In a proof-of-principle test, viral samples were prepared by extracting RNA from healthy and infected fish samples and subjected to tetra-primer PCR for simultaneous amplification of SJNNV and RGNNV genotypes. Application of PCR products on functional dual lateral flow biosensor allowed detection of the genotype of the present virus by naked eye (visual). Knowledge of the correct nodavirus genotype is a valuable tool allowing more effective diagnosis and treatment of disease pathologies.

## 2. Experimental Section

### 2.1. Oligonucleotides

Synthesis of the oligonucleotides used in the present study was performed by Eurofins Genomics AT (Vienna, Austria). The primers were designed with respect to the publicly available RGNNV and SJNNV genotype sequences (GenBank accession numbers: Y08700.1 and NC_003449.1, resp.), as described before [17, 27]. The degenerate primers UpExtNdv (5′-ACACCTGA(A/G)GA(G/C)AC(T/C) ACCGCTCC(C/A)AT-3′) and DpExtNdv (5′-C(C/G)CCA(A/T)CTGTGAA(T/C)GTCTTGTT(A/G)AAGT(C/T) (A/G)TCCC-3′) were utilized as external primers (upstream and downstream, resp.). The upstream internal primer UpInSJNdv (5′-GATTTCGTTCCATTCTCTTG-3′) was indicative for the SJNNV genotype and labelled with the hapten digoxigenin (Dig) at the 5′ end. The downstream internal primer DpInRGNdv (5′-GATTTCGTTCCATTCTCTTG-3′) was specific for the RGNNV genotype and labelled with the hapten fluorescein (Fluor) at the 5′ end. The SJNNV-specific probe (Probe_SJNdv: 5′-AGTGTCTCCAGCTTTCTTCC-3′) and the RGNNV-specific probe (Probe_RGNdv: 5′-CCACAAATGATTTCAAGTCC-3′) were both 5′ biotinylated (B). Oligonucleotides B-dA_20_ (5′-biotin-AAAAAAAAAAAAAAAAAAAA-3′), B-dC_20_ (5′-biotin-CCCCCCCCCCCCC CCCCCCC-3′), dig-dT_20_ (5′-digoxigenin-TTTTTTTTTTTTTTTTTTTT-3′), and fluor-dG_20_ (5′-fluorescein-GGGGGGGGGGGGGGGGG GGG-3′) were used as reference oligonucleotides.

### 2.2. Reference Plasmids

Two reference plasmids, specific for each genotype (GenScript, Piscataway, NJ, USA), were used as targets for tetra-primer PCR optimization studies. The sequences of the pRGNNV and pSJNNV are described in detail in [27]. A partial sequence of RGNNV coat protein gene (295 bp) and a part of SJNNV coat protein gene (301 bp) were cloned in pUC57 by EcoRV, based on the respective reference sequences.

### 2.3. Assay Principle

**Figure 1:**
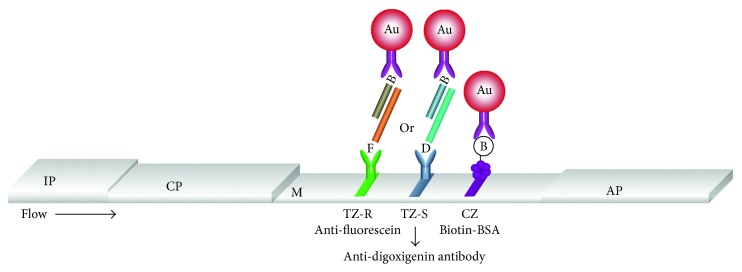
Principle of the dual nanoparticle-based lateral flow biosensor for simultaneous detection of nodavirus SJNNV and RGNNV genotypes. Two test zones (anti-fluorescein antibody (TZ-R) and anti-digoxigenin antibody (TZ-S)) and a control zone (biotinylated BSA (CZ)) have been deposited on the diagnostic membrane. The sample, containing the respective genotype of the target analyte (tetra-primer PCR product with fluorescein (F) for RGNNV genotype or digoxigenin (D) for SJNNV genotype), is hybridized with a biotinylated genotype-specific oligonucleotide probe and applied on the conjugation pad, where functionalized gold nanoparticles (Au) with anti-biotin antibody have already been added. Following that, the biosensor is immersed in the developing buffer, the sample and the nanoparticles are immobilized on the appropriate test zone, and the positive signal is visible by the naked eye, as a red zone. The excess nanoparticles bind to the control zone of the biosensor. The image shows a side view of the lateral flow biosensor. IP: immersion pad; CP: conjugation pad; M: diagnostic membrane; AP: absorbent pad. The assay components are not in scale.

The principle of the dual lateral flow biosensor is illustrated schematically in Figure 1. The genotype-specific PCR for RGNNV- and SJNNV-specific amplification products has been described in detail in [27]. Briefly, total RNA isolated from fish samples was subjected to reverse transcription reaction and a single PCR with two sets of primers (tetra-primer PCR) was performed with the produced cDNA. Tetra-primer PCR consisted of phase I, where the external primer set amplify a segment that spans the highly variable genomic region of interest, and phase II, where a lower annealing temperature is applied and the inner primers (genotype-specific primers) anneal to opposite strands. The inner primers pair off with the external primers to guide a bidirectional amplification that uses the long PCR product as a template and generates short genotype-specific fragments, although amplification of the long product continues to some degree. The inner primers were designed with a digoxigenin or a fluorescein moiety at their 5′ end; thus, the short products were labelled with digoxigenin for the SJNNV genotype or fluorescein for the RGNNV genotype. The amplified DNA hybridized in solution with the genotype-specific probes SJNNV and RGNNV, which were labelled at their 5′ end with biotin, comprising a segment complementary to their respective target. The mixture was applied to the conjugate pad of the biosensor, which was then immersed into the developing solution. The solution migrated along the LFB by capillary action and rehydrated the anti-biotin-conjugated gold nanoparticles. The hybrids were captured from immobilized anti-digoxigenin or anti-fluorescein at the respective test zone (TZ-S/TZ-R) of the biosensor and interacted with the biotinylated probes. As a result, there was accumulation of gold nanoparticles and generation of a characteristic red line at the proper test zone of the biosensor. The excess nanoparticles were captured from immobilized biotinylated BSA at the control zone of the LFB, hence generating a red line that confirmed the proper function of the biosensor. The biosensor detects only the short, genotype-specific PCR products and not the long ones. The latter hybridizes to both probes but is not captured at the test zones since it lacks a labelled end (the outer primers are unmodified). The genotype was assigned by the results of the LFB. The presence of an anti-fluorescein red zone (TZ-R) and absence of an anti-digoxigenin red zone indicated the RGNNV genotype. The SJNNV genotype was characterized by a red zone of anti-digoxigenin (TZ-S). Theoretically, presence of both genotypes in a sample would result in red zones for both immobilized antibodies.

### 2.4. Preparation of Antibody-Conjugated Gold Nanoparticles

Gold nanoparticle (Au NP) functionalization with anti-biotin antibody was performed following the previously described protocol [34]. Briefly, 1 mL of gold nanoparticles solution (30 nm) (Sigma-Aldrich, Steinhem, Germany) was adjusted to pH 9 with addition of the appropriate amount (∼25 *μ*L) of borax solution (200 mM) (AppliChem, Darmstadt, Germany). Anti-biotin antibody (4 *μ*g; Sigma-Aldrich, Steinhem, Germany) was diluted in 200 *μ*L of borax solution (20 mM) and was mixed with the Au NP solution, with gradual addition by stirring (4 times × 50 *μ*L). Following incubation at room temperature for 45 min, 100 *μ*L of 10% bovine serum albumin (BSA-AppliChem, Darmstadt, Germany) diluted in borax solution (20 mM) were added, and the final mixture was incubated at room temperature (10 min). The excess of reagents was removed by centrifugation (4500 ×g, 1 hr). The resulting pellet was redispersed, and the wash solution (1 mL 1% BSA in a 2 mM borax solution) was added. The supernatant was discarded after centrifugation (4,500 ×g, 10 min). Finally, the red pellet was redispersed in 100 *μ*L of an aqueous storage solution (0.1% BSA and 0.1% NaN_3_ in 2 mM borax).

The preparation of anti-BSA gold nanoparticles, as described in [35], was as follows: 1 mL aliquots of Au NP solutions (5 and 10 nm, Sigma-Aldrich, Steinhem, Germany) were adjusted to pH 9 with addition of 200 mM borax. A solution containing 4 *μ*g of anti-BSA antibody (Sigma-Aldrich, Steinhem, Germany) diluted in borax (20 mM) was added to each gold solution followed by stirring, and then the mixtures were incubated at room temperature for 45 min. Human serum albumin (HSA 10%, Sigma-Aldrich, Steinhem, Germany) in 20 mM borax was added in the mixture, which was incubated at room temperature (10 min). The excess reagents were removed by centrifugation (3500 ×g for 60 min), and the pellet was redispersed in 1 mL wash solution (1% HSA in 2 mM borax). After centrifugation (3500 ×g, 10 min), the supernatant was discarded, and the red pellet was redispersed in 100 *μ*L of storage solution (0.1% HAS, 0.1% NaN_3_, 2 mM borax). All incubations were carried out in the dark.

The antibody-gold nanoparticle conjugates were stored at 4°C.

### 2.5. Preparation of the Dual Lateral Flow Biosensor

The dual dry reagent lateral flow biosensors (4 × 60 mm) were prepared as described before in [34]. The biosensors' parts are positioned on a plastic adhesive backing as follows: A nitrocellulose diagnostic membrane (M: HF240MC100, 25 mm in length; Millipore, Billerica, MA, USA) was placed on a laminated card by the manufacturer. A glass fiber conjugate pad (CP: GFCP000800, 8 mm; Millipore, Billerica, MA, USA) is added below the membrane, a cellulose immersion pad (IP: CFSPOO1700, 17 mm; Millipore, Billerica, MA, USA) is positioned below the conjugate pad, and a cellulose absorbent pad (AP: same as the immersion pad) is placed just above the membrane. Each pad is overlapping its adjacent pads (∼2 mm) to make certain that the solution will migrate through the biosensor. The construction of the two test zones and the control zone was done utilizing the TLC applicator, Linomat 5, and the WinCats software (Camag, Muttenz, Switzerland). The zones were formed by loading anti-fluorescein antibody (TZ-R: polyclonal anti-fluorescein antibody; monoclonal anti-fluorescein antibody), anti-digoxigenin antibody (TZ-S: Roche Diagnostics, Mannheim, Germany), and biotinylated BSA (bBSA: Thermo Fisher Scientific Inc., Rockford, IL, USA) on the membrane and were located at 10, 15, and 20 mm distance from the edge of the membrane, respectively. In details, for the TZ-R zone, a solution consisting of 500 mg/L anti-fluorescein antibody, 50 mL/L methanol, and 20 g/L sucrose in freshly prepared 100 mM NaHCO_3_ buffer (pH 8.5) was loaded at a density of 500 ng per LFB. For TZ-S zone, a solution containing 500 mg/L anti-digoxigenin antibody, 50 mL/L methanol, and 20 g/L sucrose in 100 mM NaHCO_3_ buffer (pH 8.5) was loaded at a density of 500 ng per 4 mm membrane. Finally, for the control zone, a solution consisting of 4 g/L bBSA, 50 mL/L methanol, and 20 g/L sucrose in PBS (PBS: 0.14 M NaCl, 2.7 mM KCl, 10 mM sodium phosphate, and 1.7 mM potassium phosphate, pH 7.4) was loaded at a density of 1.6 *μ*g per 4 mm membrane. The membrane was dried in an oven for 1 h at 80°C, and the sensors were assembled as described above. All biosensors were cut (4 mm width) utilizing a Guillotine cutter and stored dry at room temperature.

### 2.6. Fish Samples, RNA Extraction, and cDNA Preparation

All samples used in the present study were European sea bass (*Dicentrarchus labrax*). One fish which was infected with nodavirus was collected from a sea-cage fish farm in Epidavros (Saronikos Gulf). Healthy fishes were reared (8 months) in experimental facilities of the Hellenic Centre for Marine Research (Athens, Greece), and used as negative controls. Fish retinas were isolated using aseptic techniques, transferred in sterile tubes, and stored at −80°C until use.

The RNeasy Mini kit (Qiagen, Hilden, Germany) was used for total RNA extraction, according to the manufacturer's instructions. Measurements of the absorbance at 260 nm (*A*_260_) with a Nanodrop 1000 spectrophotometer (Thermo Fisher Scientific, Delaware, USA) confirmed that the isolated RNA was pure while it also extrapolated its concentration.

The purified total RNA was reverse transcribed (RT) with Superscript II reverse transcriptase (Invitrogen, Carlsbad, CA). The RT reaction consisted of 0.5 mM dNTPs (dNTPs: dATP, dTTP, dCTP, dGTP; HT Biotechnology, Cambridge, UK), 2.5 *μ*M dT_20_ oligonucleotide, RNase-free H_2_O and extracted total RNA (100 ng). The reaction mixture was incubated at 65°C (5 min), quickly chilled on ice (0°C, 1 min), followed by addition of the first-strand buffer (1x), dithiothreitol (0.1 M), and RNase OUT RNase inhibitor (40 U; Invitrogen, Carlsbad, CA) and incubation at 42°C (2 min). The enzyme SSII RT (200 units) was added, and the final mixture was incubated at 42°C (50 min). The enzyme was deactivated by heating the mixture at 70°C (15 min), and the produced cDNA was stored at −20°C.

### 2.7. Tetra-Primer PCR for Nodavirus Genotyping

The tetra-primer PCR amplification was performed with GoTaq Flexi DNA polymerase (0.625 units; Promega, WI, USA) in GeneAmp PCR System 9700 cycler (Applied Biosystems, NY, USA). The reaction mixtures contained 1 × GoTaq Flexi Buffer, 200 *μ*M of each dNTP, 0.75 mM MgCl_2_, 2 *μ*L of cDNA or 5 ng of reference plasmid, 0.25 *μ*M of each of UpExtNdv and DpExtNdv primers, and 1 *μ*M of each of Dig-UpInSJNdv and Fluor-DpInRGNdv primers, in 25 *μ*L final volume. The reactions' cycling conditions were incubation at 95°C (5 min), followed by the first phase of tetra-primer PCR (10 cycles of 94°C (15 s), 60°C (30 s), 72°C (30 s)), and the second phase of amplification (30 cycles of 94°C (15 s), 50°C (30 s), 72°C (30 s)). After completion of the cycles, the mixture was incubated at 72°C (7 min) and cooled to 4°C. The absence of contamination was confirmed by addition of negative controls (containing water instead of cDNA) in each PCR series.

### 2.8. Dual Lateral Flow Biosensor Detection Assay of Reference Oligonucleotides

Four reference oligonucleotides were utilized as target sequences: oligonucleotides B-dA_20_ and B-dC_20_ were designed to contain a biotin molecule in their 5′ end, in order to interact with Au NPs functionalized with anti-biotin antibody. The oligonucleotide dig-dT_20_ was designed with a digoxigenin molecule in its 5′ end in order to be immobilized by the anti-digoxigenin antibody (TZ-S zone) while the oligonucleotide fluor-dG_20_ was designed with a fluorescein molecule in its 5′ end to interact with immobilized anti-fluorescein antibody (TZ-R zone). Two target mixtures were prepared: Dig-mixture consisted of 1 pmol B-dA_20_, 1 pmol dig-dT_20_, and ddH_2_O; Fluor-mixture consisted of 1 pmol B-dC_20_, 1 pmol fluor-dG_20_, and ddH_2_O. The mixtures were denatured at 95°C, for 3 min, and left to hybridize at 37°C for 10 minutes. Five microlitres of each target mix was applied on the LFBs. Next to them, 10 *μ*L of anti-biotin Au NPs was applied, and the LFBs were dipped in the developing solution (60 mL/L glycerol, 10 g/L SDS in PBS, and 10 mL/L Tween-20, pH 7.4). The signal was visible after 20 min. After completion of the assay, the LFBs were scanned with a desktop scanner (HP Scanjet G4050, HP, California, USA), and the band densities were quantified with ImageJ software [36].

### 2.9. Optimization of the Dual Lateral Flow Biosensor Preparation

#### 2.9.1. Anti-Fluorescein Test Zone Construction with Monoclonal or Polyclonal Anti-Fluorescein Antibody

For the TZ-R zone with monoclonal anti-fluorescein antibody, a solution consisting of 500 mg/L anti-fluorescein antibody (Millipore, Billerica, MA, USA), 50 mL/L methanol, and 20 g/L sucrose in freshly prepared 100 mM NaHCO_3_ buffer (pH 8.5) was loaded at a density of 500 ng per LFB. For the TZ-R zone with polyclonal anti-fluorescein antibody, 500 mg/L anti-fluorescein antibody (Meridian, Memphis, TN, USA) were mixed with the abovementioned buffer and loaded at a density of 500 ng per LFB. The procedures were performed as described in Section 2.5.

#### 2.9.2. Test Zone Construction with Various Amounts of Anti-Digoxigenin and Anti-Fluorescein Antibodies

In order to perform the antibody amount optimization studies, two mixes were prepared for each amount; that is, for TZ-S zone construction, two solutions were prepared: Solution 1 contained 250 mg/L while solution 2 contained 500 mg/L of anti-digoxigenin antibody diluted in 50 mL/L methanol and 20 g/L sucrose in 100 mM NaHCO_3_ buffer (pH 8.5). The TZ-R zone construction was tested with solution 3, consisting of 75 mg/L of polyclonal anti-fluorescein antibody or 500 mg/L of the same antibody (solution 4) in 50 mL/L methanol, and 20 g/L sucrose in freshly prepared 100 mM NaHCO_3_ buffer (pH 8.5).

### 2.10. Dual Lateral Flow Biosensor Detection Assay of Reference Oligonucleotides with Signal Enhancement with Gold Nanoparticle Conjugates

The target mixtures were prepared as before (Section 2.8). Five microlitres of each target mix was added to the biosensors' conjugation pad, where 5 *μ*L of Au NPs functionalized with anti-biotin was already placed. The LFBs were immersed into the developing solution (250 *μ*L), and 5 min later, 5 *μ*L of Au NPs functionalized with anti-BSA antibody was added to the conjugation pad. The biosensors were redipped into the developing solution, and the assay was completed within 20 min. Finally, the biosensors were scanned with a desktop scanner, and the band densities were quantified with ImageJ software.

### 2.11. Dual Lateral Flow Biosensor Detection Assay of Nodavirus Tetra-Primer PCR Products

Detection of the nodavirus tetra-primer PCR products was performed as follows: aliquots of PCR solutions were mixed with 90 mM NaCl, 1 pmol of each biotin-labelled genotype-specific nodavirus probe and 1 × PCR buffer (final volume: 5 *μ*L). The mixtures were incubated at 95°C (3 min), followed by hybridization (10 min, 25°C). The resulting mixtures were added to the conjugation pad, where 10 *μ*L of Au NPs functionalized with anti-biotin antibodies was already placed. The biosensors' immersion pads were then dipped in tubes containing the developing solution (250 *μ*L). The visual detection was completed within 20 min. Longer times did not affect the assay results. After completion of the assay, the LFBs were scanned with a desktop scanner, and the ImageJ software was utilized for band density quantification.

### 2.12. Optimization of the Dual Lateral Flow Biosensor Detection Assay

The dual LFB assay for tetra-primer PCR RGNNV- and SJNNV-specific product detection was optimized by comparing the detection specificity and efficiency obtained, using (i) different amounts of oligonucleotide detection probes (0.5–4 pmol/1 pmol of target; i.e., 0.5/1/2, and 4 pmol of probes) and (ii) different annealing temperatures (i.e., 25, 37, and 42°C). The parameter that resulted in the highest amount of specific signal in the appropriate test zone and the smallest amount of nonspecific signal was chosen as the optimum condition in each case.

## 3. Results and Discussion

### 3.1. Optimization of the Dual Lateral Flow Biosensor Preparation

The proposed dual biosensor format was developed by our research group and has been successfully exploited on pharmacogenetic studies for cytochrome c single nucleotide polymorphism genotyping, combined with oligonucleotide ligation reaction [34]. In that study, the biosensor was used for detection of the double-labelled single-stranded DNA products of a ligation reaction. In the present work, our aim was the detection of a hybridized complex between a hapten-labelled PCR product and a biotin-labelled genotype-specific oligonucleotide probe. In an effort to increase the signal generation ability of the proposed biosensor, several optimization studies regarding the LFB construction were performed. The construction of the test zones is the most critical part of the developed assay since several parameters could affect the assays' specificity and sensitivity. The factors which were studied include the use of monoclonal versus the polyclonal antibody for the anti-fluorescein test zone construction and the deposited antibody amount for both test zones. Signal formation is affected by the gold nanoparticle accumulation on the biosensor zones; therefore, the application of a signal enhancement methodology [35] was also investigated. All optimization studies were performed with reference oligonucleotide mixtures as described in Section 2.8.

#### 3.1.1. Monoclonal versus Polyclonal Anti-Fluorescein Antibody for Test Zone Construction

**Figure 2:**
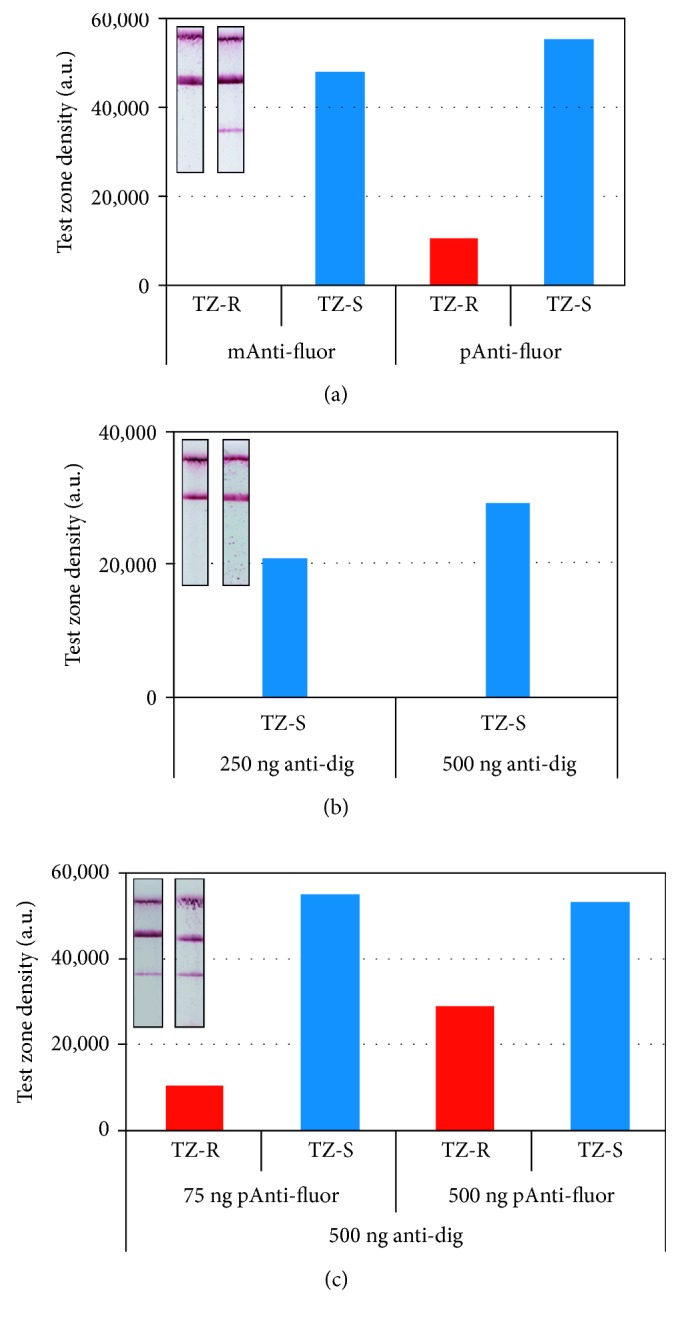
Dual lateral flow biosensor optimization studies. Representative lateral flow biosensors and signal intensity graphs for (a) effect of the use of monoclonal (mAnti-fluor) versus polyclonal (pAnti-fluor) anti-fluorescein antibody; (b) effect of the anti-digoxigenin (anti-dig) antibody amount for test zone construction; and (c) effect of the polyclonal anti-fluorescein antibody amount for test zone construction. All tests were performed with dig- and fluor-reference target mixtures. Signal is visualized with anti-biotin Au NPs. TZ-S: anti-digoxigenin zone; TZ-R: anti-fluorescein zone.

The proposed dual LFB consisted of two test lines made by anti-digoxigenin (TZ-S) and anti-fluorescein antibodies (TZ-R) and a control zone which was made by biotinylated BSA, absorbed by the membrane. The signal visualization was realized by Au NPs conjugated with anti-biotin antibodies. The anti-digoxigenin antibody performance for test zone construction was evaluated in the previously mentioned study [34], as well as other independent studies [37, 38]. Therefore, the same type of anti-digoxigenin antibody was utilized for the TZ-S construction in the present study. However, the use of the polyclonal fluorescein antibody for the construction of TZ-R zone was only evaluated in our previous study. In an attempt to increase the dual LFB specificity, a commercially available monoclonal anti-fluorescein antibody was tested in parallel with the previously used polyclonal antibody. As shown in Figure 2, the use of the monoclonal antibody did not result in any signal. Fluorescein has many isoforms, and possibly the fluorescein moiety of the utilized modified oligonucleotides could not interact with the monoclonal antibody. On the contrary, the polyclonal antibody resulted in satisfactory signal density, possibly because more antigenic epitopes were recognized in fluorescein and higher antigen amounts of antibody were immobilized; thus, more fluorescein hapten-labelled oligonucleotide was visualized.

#### 3.1.2. Antibody Amount for Test Zone Construction Effect

The immobilized anti-fluorescein and anti-digoxigenin antibody amounts were examined next. Two concentrations of each antibody were tested. The amount of anti-digoxigenin antibody on the TZ-S was initially studied (Figure 2). Use of 500 ng of antibody per 4 mm biosensor resulted in the optimum signal compared with 250 ng of antibody (1.4-fold increase). These results are in accordance with [34]. The amount of anti-fluorescein antibody was subsequently tested. The used concentrations were 75 and 500 ng of antibody per 4 mm biosensor (Figure 2). The optimum results were obtained with 500 ng of anti-fluorescein (2.9-fold increase). The use of 75 ng of the antibody resulted in a faint signal, in contrast with the results obtained in [34], possibly due to variations in the nitrocellulose membrane characteristics and additives between the two different providers.

#### 3.1.3. Effect of the Au NP Anti-Biotin Conjugate Amount and Signal Enhancing NP Complex

**Figure 3:**
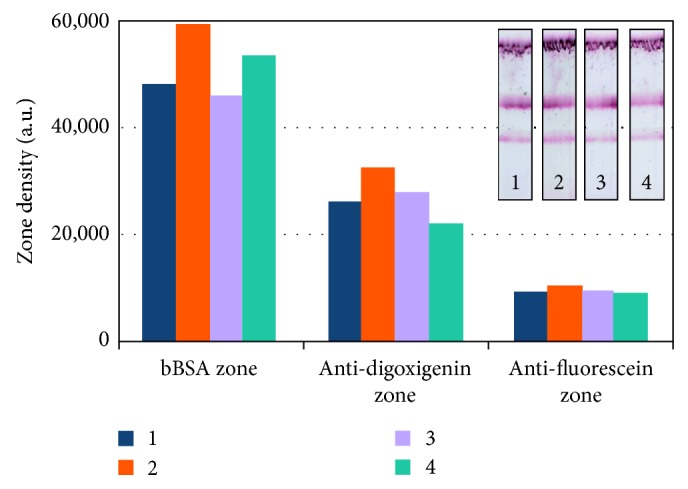
Effect of anti-biotin functionalized gold nanoparticle amount and signal enhancement with nanoparticle aggregates. Representative lateral flow biosensors and signal intensity graphs for Dig- and Fluor-reference target mixtures. Signal is visualized with (1) 30 nm gold nanoparticles functionalized with anti-biotin antibodies (5 *μ*L); (2) 30 nm Au NPs functionalized with anti-biotin antibodies (10 *μ*L); (3) 30 nm gold nanoparticles functionalized with anti-biotin antibodies (5 *μ*L) and 10 nm anti-BSA conjugated Au NPs (5 *μ*L); and (4) 30 nm gold nanoparticles functionalized with anti-biotin antibodies (5 *μ*L) and 5 nm anti-BSA conjugated NPs (5 *μ*L).

Recently, our research group developed a signal amplification methodology in one-step for nucleic acid detection lateral flow biosensors based on gold nanoparticles [35]. The “dual gold signal enhancement method” uses Au NPs functionalized with two different antibodies. The first type of Au NPs consists of nanoparticles functionalized with anti-biotin antibodies (30 nm), blocked with BSA, and the second conjugate contains anti-BSA antibody on Au NPs with different sizes (5 nm/10 nm). In that approach, the 30 nm anti-biotin conjugated Au NPs (which are used for signal generation) are forming complexes with anti-BSA conjugated Au NPs resulting in formation of large gold NP aggregates which enhance the LFB signal. Since the proposed biosensor was based on anti-biotin conjugated gold nanoparticles, the signal enhancement methodology was tested with that format. The results are presented in Figure 3. Each particle combination was tested with both Dig- and Fluor-reference mixtures. Four conjugate types were evaluated: (1) 30 nm gold nanoparticles functionalized with anti-biotin antibodies (5 *μ*L), which was, also, the basic quantity for detection; (2) 30 nm Au NPs functionalized with anti-biotin antibodies (10 *μ*L) since the final volume of the gold conjugates of the dual format was 10 *μ*L; (3) 30 nm gold nanoparticles functionalized with anti-biotin antibodies (5 *μ*L) and 10 nm anti-BSA conjugated Au NPs (5 *μ*L), and (4) 30 nm gold nanoparticles functionalized with anti-biotin antibodies (5 *μ*L) and 5 nm anti-BSA conjugated NPs (5 *μ*L). When the formulation (1) (5 *μ*L of 30 nm anti-biotin conjugated Au NPs) was used as the standard or default condition, a signal increment of both test zones was observed following the addition of 10 nm gold anti-BSA conjugate. However, the control zone signal slightly decreased. In contrast, when 5 nm gold anti-BSA conjugate was added in 5 *μ*L of 30 nm anti-biotin conjugates, test zone signal decreased, and control zone signal increased. Thus, even though the signal enhancement method increased the signal intensity with the use of 10 nm anti-BSA Au NPs, compared with Au NPs basic formulation, the use of the double quantity (10 *μ*L) of 30 nm Au NPs functionalized with anti-biotin antibodies gave more intense signals in all test and control zones. In order to keep the dual lateral flow biosensor format as simple as possible, the use of a single Au NP conjugate was preferred, and the 30 nm gold nanoparticles functionalized with anti-biotin antibodies with 10 *μ*L amount was used throughout the study.

### 3.2. Optimization of the Dual Lateral Flow Biosensor Detection Assay

#### 3.2.1. Oligonucleotide Probe Amount Effect

**Figure 4:**
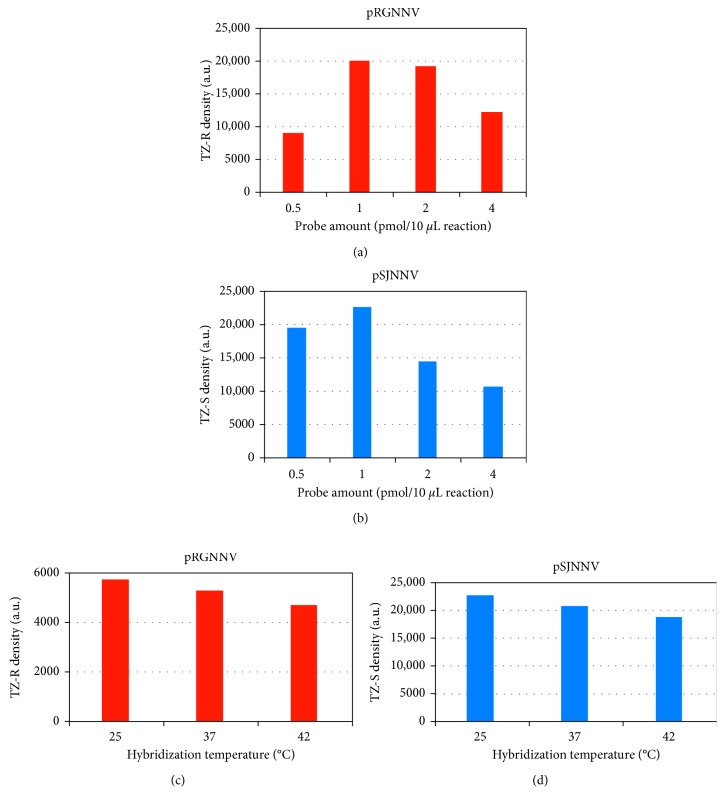
Dual lateral flow biosensor assay optimization studies. Representative lateral flow biosensors and signal intensity graphs for (a) oligonucleotide probe amount effect on plasmid pRGNNV amplification product hybridization mixture; (b) oligonucleotide probe amount effect on plasmid pSJNNV amplification product hybridization mixture; (c) hybridization temperature effect on the pRGNNV PCR product and specific oligonucleotide probe hybridization; and (d) hybridization temperature effect on the pSJNNV PCR product and specific oligonucleotide probe hybridization. The signal is visualized with anti-biotin Au NPs. TZ-S: anti-digoxigenin zone; TZ-R: anti-fluorescein zone.

Optimization studies for assessment of the oligonucleotide probe impact in the hybridization reaction mixtures were performed. The oligonucleotide probes were tested in amounts of 0.5–4 pmol/1 pmol of target (Figures 4 and 4). Both test zones resulted in optimum signals when 1 pmol of probe was used and decreased with higher amounts of probe. This observation is attributed to the fact that at high levels, the amount of biotin-labelled probe exceeds the binding capacity of the anti-biotin-functionalized nanoparticles, and the biotin-labelled probe which hybridizes to the specific target sequence competes with the unhybridized probe for binding to limited anti-biotin conjugated NPs. When higher amounts of biotinylated probes are used, the amount of nanoparticles that bind to the free probe is increasing. Even though these nanoparticles move along the LFB, they cannot be immobilized from the deposited antibodies in the test zones, and the red bands become fainter [39].

#### 3.2.2. Hybridization Temperature Effect

The hybridization temperature effect on target PCR product and specific oligonucleotide probe hybridization was tested with a temperature of 25–42°C. As observed in Figures 4 and 4, the density of both test zones is more intense at 25°C. In higher temperatures, the densities are decreasing slightly, due to inhibition of the hybridization. For that reason, the 25°C temperature was chosen for the optimum hybridization in all subsequent experiments.

### 3.3. Dual Lateral Flow Biosensor Assay Reproducibility

**Figure 5:**
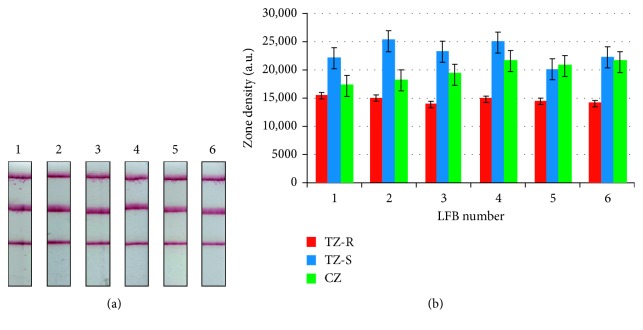
Reproducibility study. (a) Typical images of lateral flow biosensors with anti-biotin-gold nanoparticle conjugates after applying Dig- and Fluor-reference target mixtures as targets. (b) Optical density of the test and control zones. Graph of the intensities of the lateral flow biosensor test and control zones for reproducibility assessment (CV_TZ-R_: 3.9%; CV_TZ-s_: 8.2%; CV_CZ_: 9.2%, *n* = 6). TZ-R: anti-fluorescein zone; TZ-S: anti-digoxigenin zone; CZ: control zone; CV: coefficient of variation.

The dual lateral flow assay reproducibility was assessed since it is one of the most important parameters for successful biosensor development. The proposed assay reproducibility was assessed with simultaneous application of the Dig- and Fluor-reference mixtures on the dual biosensors. Six biosensors which were prepared in different batches (i.e., LFBs 1 and 2: batch 1; LFBs 3 and 4: batch 2; and LFBs 5 and 6: batch 3) were tested with the reference mixtures, and the test and control zones intensities were measured. The results are presented in Figure 5. Analysis of the TZ-R test zone of the LFBs gave a coefficient of variation (CV) of 3.9%, while analysis of the TZ-S test zone resulted in 8.2% CV. The CV for the control zone was 9.2%, indicating excellent reproducibility.

### 3.4. Proof-of-Principle Nodavirus Amplification Product Detection with Dual Lateral Flow Biosensor

**Figure 6:**
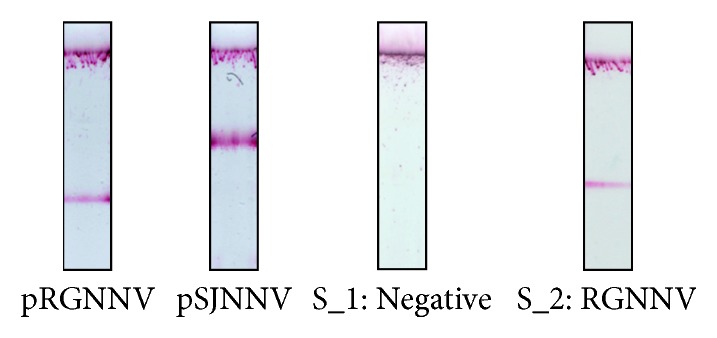
Visual detection of nodavirus genotype-specific products with dual lateral flow biosensors. Representative LFBs with amplification products of tetra-primer PCR performed with pRGNNV and pSJNNV reference plasmids and a healthy (S_1) and an infected (S_2) *D. labrax* sample.

The dual lateral flow biosensor was used to detect amplification products of both genotype-specific plasmids (pRGNNV and pSJNNV), one nodavirus infected *D. labrax* sample and one healthy (negative) *D. labrax* sample, as proof-of-principle for the proposed assay. All samples were subjected to tetra-primer PCR, and the PCR products were hybridized with the genotype-specific probes. Both probes were added in the hybridization mixture, and the resulting amplification product-probe complexes were applied on the biosensors. The present genotype was assigned by a single biosensor, and the results are shown in Figure 6. The presence of a single TZ-R zone for the pRGNNV product and a single TZ-S zone for the pSJNNV confirmed the specificity of the proposed dual LFB for each genotype. The noninfected sample did not show any signal in the test zones, correctly indicating the absence of nodavirus and further confirming the LFB specificity. The infected sample was positive with low signal intensity, and it was correctly classified as RGNNV. The genotype of the sample was previously determined by direct sequencing by an independent research group.

As mentioned in our previous studies [27, 28, 33] and independent research groups [40], there is a tremendous difficulty to obtain virus samples of various strains. The location of samples belonging to the SJNNV genotype was not feasible, and all samples previously analyzed by our research group belonged to the RGNNV genotype. Therefore, the present work was merely focused on the dual lateral flow biosensor optimization, contributing towards a fully developed nanobiosensor for nodavirus genotyping. Analysis of the plasmid tetra-primer PCR products, along with amplification products from one healthy and one nodavirus-infected sample confirmed the feasibility of the proposed biosensor. Studies for collection of a high number of fresh samples from different geographical regions, in order to obtain both nodavirus genotypes, to fully validate the proposed methodology are in progress by our research group.

## 4. Conclusions

The proposed dual lateral flow biosensor constitutes a step forward to a robust, rapid, and accurate tool for fish virus genotype assessment with ease and low cost. The assay can be utilized as a potential detection system for virus genotyping by small- and medium-size research labs and the aquaculture industry, providing the means for effective vaccine and diagnostic development. The results demonstrate the optimization studies for a rapid single-step assay, which requires low amount of the analyzed sample and provides simultaneous amplification and genotyping of nodavirus DNA in a single, closed-tube methodology. The assay was optimized in terms of the biosensors' preparation and the detection assay parameters, demonstrating attractive characteristics with respect to specificity and reproducibility. The optimum goal for the proposed methodology is to replace the costly sequencing for virus genotyping, since such simple-to-use and low-cost methods are ideal for medium-scale laboratories.

The main advantage of the proposed method compared with previously used methods (i.e., gel electrophoresis and melting analysis) is that the dual biosensor minimizes the need for specialized and costly instrumentation and reagents. Therefore, it enables rapid and low-cost genotyping of nodavirus by visual detection of the RGNNV/SJNNV amplification product. Also, the tetra-primer PCR product is directly hybridized with genotype-specific probes without prior purification from the excess of primers and dNTPs, and the hybridization mixture is applied on the biosensors' conjugate pad, minimizing the possibility for contamination. Use of the genotype-specific probe and product detection by hybridization provides extra sequence confirmation, in contrast with electrophoresis that provides only the size of the amplification products. The visual detection of the genotype-specific product is completed in 20 min, and the overall assay can provide a samples' genotype in less than 4 hours. Finally, the lateral flow biosensor format minimizes the requirements for highly qualified personnel for performing the test and interpreting the results.
